# Chronic Kidney Disease and Acute Kidney Injury Outcomes Post Left Ventricular Assist Device Implant

**DOI:** 10.7759/cureus.7725

**Published:** 2020-04-18

**Authors:** Muhammad S Ajmal, Umang M Parikh, Harveen Lamba, Carl Walther

**Affiliations:** 1 Nephrology, Baylor College of Medicine, Houston, USA; 2 Surgery, Baylor College of Medicine, Houston, USA

**Keywords:** left ventricular assist device (lvad), chronic kidney disease, heart failure, acute kidney injury

## Abstract

Introduction

Left ventricular assist devices (LVAD) are used as a bridge to heart transplant or destination therapy for patients with the New York Heart Association (NYHA) class 3 or 4 heart failure. Acute kidney injury (AKI) or need for renal replacement therapy (RRT) post-LVAD implant can lead to poor outcomes. Identifying risk factors of AKI post-LVAD implant can help stratify potential LVAD candidates.

Methods

This is a retrospective study of all patients who received continuous-flow LVAD at our institution from January 2015 until August 2017. We calculated the incidence of AKI and the need for RRT post-LVAD implant, as well as the rate of renal recovery and survival rates at 30 days and 1-year post-LVAD implant. The presence of chronic kidney disease (CKD) and proteinuria was assessed, and kidney ultrasound results were reviewed on all patients, if available. CKD was present if estimated glomerular filtration rate (eGFR) was <60 mL/min per 1.73m^2^ for ≥3 months preceding LVAD implant and/or presence of proteinuria ≥ 20 mg/dL on two or more urine samples prior to LVAD implant and/or an abnormal kidney ultrasound with increased echogenicity, small size <9 cm or scarring. AKI was defined as per the current Kidney Disease Initiative Global Outcomes (KDIGO) guidelines.

Results

A total of 137 patients received LVAD during this time period. There were 112 males and 25 females with a mean age of 59.2 years. Incidence of AKI and the need for RRT post-LVAD implant were 64% and 19.7%, respectively. Sub-group analysis was performed based on the presence of CKD, advanced CKD stage (Stage 1-2 vs 3-5), proteinuria and abnormal kidney ultrasound. The incidence of AKI post-LVAD implant was significantly higher if baseline CKD was present (*P* = 0.028), and patient had an advanced CKD stage (*P* = 0.008). The need for RRT post-LVAD implant was significantly higher if baseline CKD was present (*P *= 0.015), and the patient had an abnormal kidney ultrasound (*P *= 0.04). Thirty-day and one-year mortality rates post-LVAD implants were 4.3% and 21.1%, respectively for the entire cohort. Out of the 27 patients requiring RRT, nine (33.3%) came off RRT before one year. Compared to the eGFR on the day of LVAD implant, eGFR at 30 days post-LVAD implant was higher in 57% and lower in 42% patients. At one year, this eGFR improvement reversed and eGFR was lower in 67% and higher in 32% patients.

Conclusion

The incidence of AKI and need for RRT post-LVAD implant are very high. The presence of CKD, advanced CKD stage, and an abnormal kidney ultrasound are statistically significant risk factors of AKI post-LVAD implant and/or need for RRT. Identifying these renal risk factors can help stratify the potential LVAD candidates. Only one out of three patients requiring RRT achieved dialysis independence by one-year post-LVAD implant.

## Introduction

Heart failure remains a leading cause of morbidity and mortality worldwide with an estimated 6.5 million adults in the United States presenting with heart failure [1]. It was a contributing cause of one in eight deaths in 2017 and costs the nation an estimated $30.7 billion in 2012 [1-2]. In general, the mortality following hospitalization for patients with heart failure is 10.4% at 30 days, 22% at one year, and 42.3% at five years, despite marked improvement in medical and device therapy [3]. Drug therapy has been the cornerstone of mild-moderate heart failure with some success in severe heart failure cases [4]. Nevertheless, the survival and the quality of life of patients with severe heart failure remain limited. Other options for severe NYHA class 3-4 heart failure include cardiac transplantation which provides substantial individual benefit, but with approximately 3,500 heart transplants performed each year worldwide, more than half of which are in the US, the overall impact on disease burden is still small [5].

Left ventricular assist devices (LVAD) are an electromechanical device for assisting cardiac circulation, which is used either to partially or to completely replace the function of a failing heart. The first LVAD system was created by Domingo Liotta at Baylor College of Medicine in Houston in 1962. The first successful implantation of an LVAD was completed in 1966 by Dr. Michael E. DeBakey to a 37-year-old woman. A Para corporeal (external) circuit was able to provide mechanical support for 10 days after the surgery [6]. Since the inception of the artificial heart program at the National Institutes of Health (NIH) in 1964, various circulatory-support devices have been developed for short-term use in patients with end-stage heart failure [7]. In 1994, the Food and Drug Administration (FDA) approved pneumatically driven LVADs as a bridge to transplantation, and self-contained, vented electric devices were approved for this purpose in 1998 [8]. Since then, the ventricular assist device technology has evolved significantly from a larger pulsatile flow device (Heartmate 1) designed for temporary use to a more compact continuous flow device (Heartmate 2 and 3) that gained acceptance as a bridge to transplantation or destination therapy for patients with end stage heart failure [9-11]. Short-term use of these devices in patients awaiting transplantation normalizes hemodynamics, improves end-organ dysfunction and exercise tolerance, allows patients to be sent home, and provides a reasonable quality of life, with a relatively low incidence of major adverse events [12-14].

The kidneys receive approximately 25% of the cardiac output (about 1.0 to 1.1 liters per minute) and depend on the cardiac output to maintain enough glomerular perfusion and thus the glomerular filtration rate (GFR) [15]. Heart and kidney are closely related, and heart failure often precipitates acute kidney injury (AKI) with further worsening of volume overload and pulmonary congestion leading to the cardio-renal syndrome. The presence of underlying chronic kidney disease or a decreased renal reserve increases the risk of acute kidney injury in the setting of heart failure. LVADs are implanted to provide circulatory support by assisting the cardiac pump function. This should theoretically improve renal perfusion although possible renal hypoperfusion during the perioperative period remains a risk for developing AKI. The effects of LVAD implant on renal function including the rate of post-operative AKI and long-term effects on renal function have been reported by various centers performing LVAD placement. But most studies did not report underlying chronic kidney disease and patient’s baseline GFR in steady-state three months or more prior to the LVAD implant. As most patients have fluctuating kidney function at or around the time of LVAD implant due to severe heart failure, an assessment of their baseline renal function is not possible unless old records are available from period when the patient was not acutely sick.

We present a retrospective study from a single center reporting the association of underlying CKD and renal outcomes post-LVAD implant. We aim to identify baseline renal risk factors that can help stratify potential LVAD candidates.

## Materials and methods

We present a retrospective study reporting the incidence of AKI and the need for renal replacement therapy (RRT) in patients who received an LVAD at our institution from January 2015 to August 2017. A baseline CKD status of all patients was obtained if data available and post-LVAD implant renal outcomes were reported in correlation with the baseline CKD status. All patients received continuous-flow LVAD implant for indications of Class 3 or 4 NYHA heart failure due to ischemic or non-ischemic cardiomyopathy.

Data was obtained from chart review after appropriate IRB approval. We calculated the incidence of AKI and the need for RRT post-LVAD implant and reviewed charts for up to one-year post-LVAD implant to monitor renal outcomes. We identified renal risk factors and reviewed data prior to LVAD implant to obtain baseline kidney function of each patient. We included the presence of CKD, advanced CKD stage (Stage 3 or higher), proteinuria, and an abnormal kidney ultrasound as the renal risk factors and studied their impact on the incidence of AKI and RRT need post-LVAD implant. CKD was defined as a structural or functional abnormality of the kidney lasting ≥3 months. Proteinuria was defined as the presence of >20 mg/dL protein on urine analysis on at least 2 samples obtained prior to the LVAD implant. Abnormal kidney ultrasound (KU) included abnormal echogenicity, small kidneys <9 cm, scarring or presence of multiple complex kidney cysts. AKI was defined per current KDIGO guidelines and further categorized as Stage 1, 2, or 3 AKI [16]. Stage 1 AKI includes an increase in serum creatinine (Cr) 1.5-1.9 times from baseline or increase in serum Cr of ≥0.3 in 48 h. Stage 2 AKI includes an increase in serum Cr 2.0-2.9 times from baseline, and stage 3 AKI includes serum Cr increase ≥3 times from baseline or need for RRT.

Data was kept secure and de-identified. Statistical analysis of the data was done using Chi-square test. A P-value of ≤0.05 was considered statistically significant.

## Results

A total of 137 patients received LVAD implant during the specified time period. There were 112 male and 25 female patients with a mean age of 59.2 years. Racial distribution included 63 Caucasians, 38 African Americans, 29 Hispanics, and five Asians. Patients were divided into two main groups based on the presence or absence of underlying CKD, and sub-group analysis was done separately for renal risk factors including advanced CKD stage (stage 3 or higher), proteinuria, and presence of an abnormal kidney ultrasound. The baseline characteristics of the patients, including in our study, are reported in Table 1.

**Table 1:** Baseline characteristics of patients who received LVAD implant stratified into two groups based on the presence of CKD LVAD, left ventricular assist device; GFR, glomerular filtration rate; CKD, chronic kidney disease; COPD, chronic obstructive pulmonary disease; CABG, coronary artery bypass grafting

Baseline Characteristics	CKD group	No CKD group
Age	61 ± 12 years	56 ± 14 years
Sex	82% Male, 18% Women	79% Male, 21% Women
Baseline GFR ≥ 3 months	53 ± 21 ml/min/1.73m²	86 ± 16 ml/min/1.73m²
Left ventricular ejection fraction	21 ± 5%	21 ± 6%
INTERMACS profile mean	2	3
Body mass index	28.6	29.1
Smokers	34(40%)	28(66%)
Hypertension	69(82%)	28(66%)
Diabetes mellitus	46(54%)	17(40%)
COPD	16(19%)	7(17%)
Cerebrovascular accident	26(22%)	10(24%)
Peripheral vascular disease	5(6%)	2(5%)
History of myocardial Infarction	21(25%)	10(24%)
History of CABG	20(23%)	8(19%)
Hemoglobin	11.22 g/dl	12.04 g/dL
Platelets	186.9	217.4

Patients in the CKD group were slightly older than the non-CKD patients. Male to female ratio was comparable. CKD group had higher burden of comorbidities including hypertension, diabetes mellitus and history of coronary artery bypass surgery. They had slightly lower mean hemoglobin and platelet counts. CKD group patients had a poor INTERMACS profile which represents a sicker state. INTERMACS profile provides a general description of the patients receiving LVAD or heart transplantation [17]. INTERMACS 2 profile represent a steady decline state with a patient who has been demonstrated “dependent” on inotropic support but nonetheless shows signs of continuing deterioration in nutrition, renal function, fluid retention, or other major status indicator. INTERMACS profile 3 describe a stable but inotrope dependent state after repeated documentation of failure to wean without symptomatic hypotension, worsening symptoms, or progressive organ dysfunction.

Overall incidence of AKI in LVAD recipients was 64.3% including all stages. RRT (either continuous veno-venous hemodialysis or conventional hemodialysis) was required in 19.7% patients post-LVAD implant. Sub-group analysis was performed based on the presence of underlying renal risk factors to see if incidence of AkI or need for RRT post-LVAD implant differ significantly (Table 2).

**Table 2:** Incidence of AKI and RRT in all patients and subgroups based on presence of renal risk factors AKI, acute kidney injury; RRT, renal replacement therapy; US, ultrasound

	Incidence of AKI	P-value	Incidence of RRT	P-value
All patients	88/137=64%		27/137=19.7%	
CKD group	64/84=76%	0.03	23/84=27%	0.015
No CKD group	18/42=43%		4/42=9%	
CKD (Stage 1-2)	15/25=60%	0.008	6/25=24%	0.78
CKD (Stage 3-5)	41/47=87%		11/47=23%	
Proteinuria	33/45=73%	0.078	10/45=22%	0.53
No proteinuria	49/85=58%		15/85=18%	
Normal kidney US	63/103=61%	0.4	16/103=15%	0.045
Abnormal kidney US	17/24=71%		8/24=33%	

Baseline CKD status was available for 126 patients. Out of those, 84 patients had CKD present and 42 patients had no underlying CKD. In the CKD present group (*N* = 84), a total of 64 patients had AKI post-LVAD implant and 23 patients required RRT. The incidence of AKI and the need for RRT were 76 % and 27%, respectively. In the no underlying CKD group (*N *= 42), a total of 18 patients developed AKI and four needed RRT. Thus, incidence of AKI was 43% and need for RRT was 9%. The difference between the incidence of AKI post-LVAD implant in CKD and no CKD groups was statistically significant (*P *= 0.028). Similarly, the difference between the incidence of RRT need post-LVAD implant in CKD and no CKD groups was also statistically significant (*P *= 0.0153).

We further divided patients based on the baseline CKD stage. Patients with underlying CKD 1 and 2 stage (*N *= 25) had incidence of AKI 60% (*N *= 15) and RRT needed in 6 (24%) patients. Patients with CKD 3 (*N *= 42) had incidence of AKI 88% (*N *= 37) and RRT needed in 8 (19%) patients. Patient with CKD 4 (*N *= 5) had an incidence of AKI 80% (*N *= 4) and RRT was needed in 4 (80%) patients. There was only one patient with underlying CKD 5/ESRD who was on maintenance dialysis prior to admission and he required RRT. 11 patients had CKD based on the presence of proteinuria or an abnormal kidney ultrasound but did not have eGFR results available from ≥ 3 months prior to LVAD implant and thus were excluded from the CKD staging sub-group analysis. Compared to CKD 1-2, a higher CKD stage 3-5 was a statistically significant risk factor for AKI post LVAD implant (*P *= 0.008). However, compared to CKD 1-2, a higher CKD stage 3-5 was not a statistically significant risk factor for RRT need post-LVAD implant (*P *= 0.78).

Proteinuria was present in 45 (33%) patients and absent in 85 (62%) patients. 7 (5%) patients had no prior proteinuria assessment available. In the patient with no proteinuria, AKI post-LVAD implant occurred in 49 (58%) patients and 15 (17.6%) patients required RRT. 1 patient was initiated on RRT prior to LVAD implant. In the patient with proteinuria, AKI post-LVAD implant occurred in 33 (73%) patients and 10 (22.2%) patients required RRT. The presence of proteinuria was not a statistically significant risk factor for AKI post-LVAD implant (P=0.078). Similarly, the presence of proteinuria was not a statistically significant risk factor for RRT need post-LVAD implant (*P *= 0.5288).

Kidney ultrasound results were reviewed for all patients. 103 patients had normal kidney ultrasounds and 24 had abnormal results. 10 patients did not get a kidney ultrasound prior to LVAD implant. In patients with normal kidney ultrasound, 63 (61%) patients had AKI post-LVAD implant and 16 (15.5%) patients required RRT. 1 patient was initiated on RRT prior to LVAD implant. In patients with an abnormal kidney Ultrasound, 17 (71%) patients suffered AKI, and eight (33%) patients required RRT. The presence of an abnormal kidney ultrasound was not a statistically significant risk factor for AKI post-LVAD implant (*P* = 0.376), but the presence of an abnormal kidney ultrasound was a statistically significant risk factor for RRT need post-LVAD implant (*P* = 0.0448).

Mortality and Dialysis Independence Post-LVAD Implant

**Figure 1:**
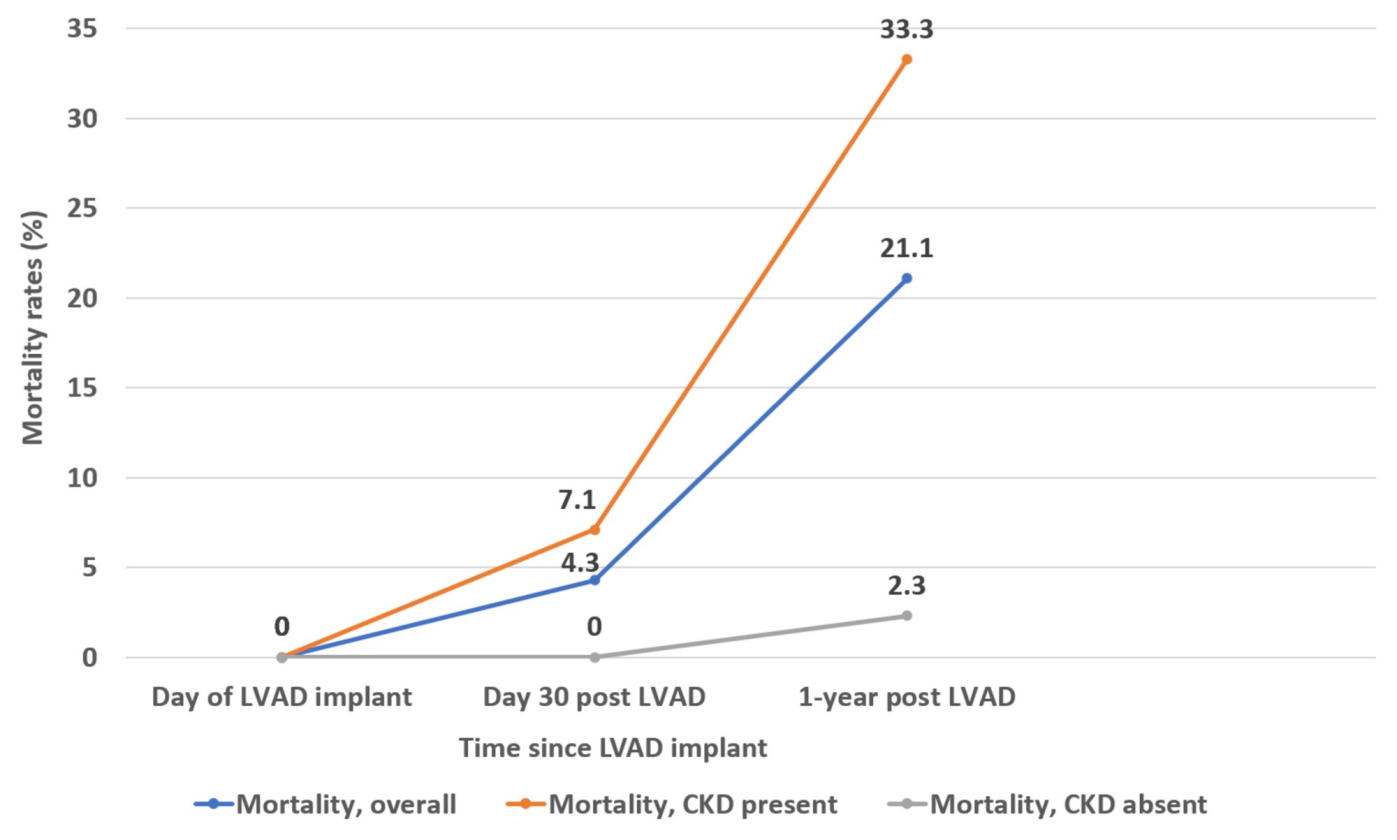
Morality rates post-LVAD implant and effect of CKD on mortality rates LVAD, left ventricular assist device; CKD, chronic kidney disease

All LVAD recipients were followed for up to 1-year post implant and 30 day and 1-year mortality rates post-LVAD implant were calculated. For those patients who required RRT post LVAD-implant, the rate of dialysis freedom/renal recovery was calculated at 1-year post implant. (Table 3). Mortality rate at 30 days and 1-year post LVAD implant was 4.3% and 21.1% respectively for the entire cohort. The mortality rate was higher if underlying CKD was present and this difference was statistically significant at 1-year post-LVAD implant (*P *= 0.0001; Table 3; Figure 1).

**Table 3:** Morality rates, eGFR change, and dialysis independence post LVAD implant CKD, chronic kidney disease; eGFR, estimated glomerular filtration rate; LVAD, left ventricular assist device

Outcomes	30 Days post LVAD	1-year post LVAD	P-Value
Mortality, Overall	6/137=4.3%	29/137=21.1%	
Mortality, CKD present	6/84=7.1%	28/84=33.3%	P=0.0001
Mortality, CKD absent	0/42=0%	1/42=2.3%	
eGFR changes in LVAD recipients	Increased 57% Decreased 42%	Increased 32% Decreased 67%	
Dialysis independence, Overall	Reported at 1 year	9/27=33%	
If CKD present		5/23=22%	
If CKD absent		4/4=100%	

**Figure 2:**
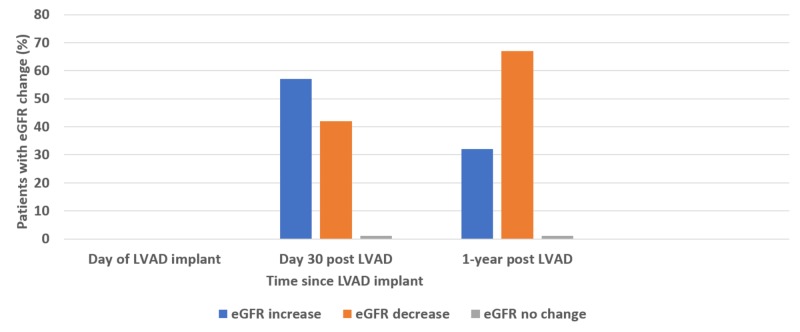
Patients with eGFR change post-LVAD implant at 30 days and one year eGFR, estimated glomerular filtration rate; LVAD, left ventricular assist devices

Dialysis independence was defined as recovery of renal function to a level that RRT was not required anymore. This was reported for all patients requiring RRT at one-year post-LVAD implant. Out of the 27 patients requiring RRT post-LVAD implant in all cohort, only nine (33%) recovered renal function and came off RRT before one year. In patients with underlying CKD who required RRT post-LVAD implant, only 5/23 (22%) achieved dialysis independence by one year. However, all 4 patients requiring RRT post-LVAD implant with no underlying CKD achieved dialysis independence before one year. eGFR at 30-days post-LVAD implant was higher in 57% of the patients but this effect faded and by 1-year post-LVAD implant 67% patient had lower eGFR compared to their pre-LVAD eGFR baseline (Figure 2).

## Discussion

Incidence rates of AKI and the need for RRT post-LVAD implant were very high per our retrospective data. These findings are similar to previously reported data. Muslem R *et al*. reported a multicenter retrospective review of 241 patients who received continuous-flow LVAD. AKI criteria were met in 169 (70%) patients, of whom 109 (45%) were in AKI Stage I, 22 (9%) in Stage II, and 38 (16%) in Stage III. One-year mortality rates in patients without AKI and AKI Stages I, II, and III were 18.7%, 26.4%, 23%, and 51%, respectively (log rank, *p* = 0.001) [18]. Harmon DM *et al.* conducted a retrospective review of 246 LVAD recipients and reported that 68 (28%) patients developed moderate/severe AKI [19]. LVAD candidates consist of patients with end-stage heart failure with frequent episodes of hemodynamic instability leading to changes in renal perfusion. This AKI is considered potentially reversible once the cardiac output improves post implant. Hasin T *et al.* showed that eGFR post-LVAD implant improved during the 1st month and remained above the pre-LVAD value even at 3 and 6 months [20]. Surgical LVAD placement initially increases the risk of AKI due to a peri-operative decrease in renal perfusion. This is followed by an improvement in the renal perfusion and thus the eGFR. This improvement in the eGFR by one month has been shown to be associated with 31% reduction in mortality but not re-admission [21].

Due to the tenuous hemodynamic status and frequent AKI episodes around the time of LVAD implant, often an assessment of baseline kidney function or renal reserve is difficult. Most of the studies reporting renal outcomes in LVAD recipients did not provide information about the baseline renal function of the patients in steady state. Our study was different as we reported the baseline renal function of the patients by retrospectively reviewing the old records of each patient for at least 3 or more month prior to the date of LVAD implant. We identified renal risk factors including the presence and severity of CKD, proteinuria, and structural kidney abnormalities and analyzed their association with renal outcomes post-LVAD implant. Our results showed that the presence of CKD, advanced CKD (Stage 3 or higher) and an abnormal kidney ultrasound significantly increase the risk of AKI and/or need for RRT post-LVAD implant. The presence of underlying CKD was associated with a significant increase in mortality rates at 30 days and 1-year post-LVAD implant.

An interesting observation was noted in our study regarding the eGFR trend post-LVAD implant. eGFR increase at 30-day post-LVAD implant was consistent with other reported studies, but the effect reversed by one year, and 67% patients had a lower eGFR at that point. This effect could be due to continued worsening of the native cardiac function or an adverse effect of the pulsatile flow generated by the LVAD and also reported with the newer continuous-flow LVAD [22]. Recovery of renal function after AKI Stage 3 requiring RRT remains rather low. At one year post LVAD implant, two-thirds of the patients were still requiring renal replacement therapy. Again, the presence of underlying CKD prior to LVAD implant was a significant risk factor and, in our study, all four patients who required RRT post-LVAD implant but did not have underlying CKD achieved dialysis independence before completion of the first year post LVAD implant. This strongly suggests that stratifying the LVAD candidates based on the renal risk factors described in our study help identify patients who are at high risk of mortality and poor renal outcome.

## Conclusions

Incidence rates of AKI and the need for RRT remain very high after LVAD implantation. The presence of CKD, advanced CKD stage, and an abnormal kidney ultrasound are statistically significant risk factors of AKI post-LVAD implant and/or need for RRT. Underlying CKD is associated with higher mortality and a lower chance of achieving dialysis independence if AKI requiring RRT develop post-LVAD implant. Identifying these renal risk factors can help stratify the potential LVAD candidates.
